# The Effect of Improved Water Supply on Diarrhea Prevalence of Children under Five in the Volta Region of Ghana: A Cluster-Randomized Controlled Trial

**DOI:** 10.3390/ijerph121012127

**Published:** 2015-09-25

**Authors:** Seungman Cha, Douk Kang, Benedict Tuffuor, Gyuhong Lee, Jungmyung Cho, Jihye Chung, Myongjin Kim, Hoonsang Lee, Jaeeun Lee, Chunghyeon Oh

**Affiliations:** 1 Korea International Cooperation Agency, 825 Daewangpangyo-ro, Sujeong-gu, Seongnam-si, Gyeongo-do 13449, Republic of Korea; E-Mails: jesuscha@koica.go.kr (S.C.); ghlee@koica.go.kr (G.L.); jmcho@koica.go.kr (J.C.); serenti@koica.go.kr (M.K.); hsl810@koica.go.kr (H.L.); jelee21@koica.go.kr (J.L.); 2 World Vision Korea, 77-1, Yeouinaru-ro, Yeongdeungpo-gu, Seoul, 07327, Republic of Korea; E-Mails: douk_kang@wvi.org (D.K.); jeehae_chung@wvi.org (J.C.); 3 Training Research and Networking for Development, Post Office. Box Cantonments 6135, Cantonments, Accra, Ghana; E-Mail: btuffuor@gmail.com; 4 Department of Disease Control, Faculty of Infectious and Tropical Disease, London School of Hygiene & Tropical Medicine, Keppel Street London WC1E 7HT, London, UK

**Keywords:** improved water supply, diarrhea, children under five, Ghana, cluster-randomized controlled trial

## Abstract

Although a number of studies have been conducted to explore the effect of water quality improvement, the majority of them have focused mainly on point-of-use water treatment, and the studies investigating the effect of improved water supply have been based on observational or inadequately randomized trials. We report the results of a matched cluster randomized trial investigating the effect of improved water supply on diarrheal prevalence of children under five living in rural areas of the Volta Region in Ghana. We compared the diarrheal prevalence of 305 children in 10 communities of intervention with 302 children in 10 matched communities with no intervention (October 2012 to February 2014). A modified Poisson regression was used to estimate the prevalence ratio. An intention-to-treat analysis was undertaken. The crude prevalence ratio of diarrhea in the intervention compared with the control communities was 0.85 (95% CI 0.74–0.97) for Krachi West, 0.96 (0.87–1.05) for Krachi East, and 0.91 (0.83–0.98) for both districts. Sanitation was adjusted for in the model to remove the bias due to residual imbalance since it was not balanced even after randomization. The adjusted prevalence ratio was 0.82 (95% CI 0.71–0.96) for Krachi West, 0.95 (0.86–1.04) for Krachi East, and 0.89 (0.82–0.97) for both districts. This study provides a basis for a better approach to water quality interventions.

## 1. Introduction

Globally, an estimated 633 million people lack access to safe water sources, 319 million of whom live in sub-Saharan Africa, and 2.4 billion of whom do not use improved sanitation, as defined by the WHO/UNICEF Joint Monitoring Programme [1]. Despite substantial progress made during the last decades, 11% of the global population are still not drinking improved water and only 64% are able to access improved sanitation [1]. Diarrhea is the main killer of children, estimated to have killed 558,000 children aged 1−59 months in 2013 [2].

Diarrhea is also a leading cause of morbidity and mortality in Ghana [3], with an estimated diarrhea-specific mortality of 7% and a prevalence of 20% [4] in children under five years of age. Although little published research is available on the epidemiology of diarrheal illness in Ghana, a lack of access to reliable, clean drinking water is likely a key factor in making diarrheal illness a leading cause of morbidity, particularly among children.

Although a number of studies have been conducted to explore the effect of water quality improvement, the majority of them [5,6,7,8,9,10,11,12,13,14,15] mainly focused on point-of-use water treatment. The interventions for improving water quality could be performed at the household level or at the water source level. Recent systematic reviews [16,17,18,19] have reported on three to eight trials or controlled before-and-after studies of improved water supplies, which have shown that most of the studies were based on quasi-randomized controlled trials. Furthermore, other studies [20,21,22,23,24,25,26,27,28] investigating the effect of improved water supply were based on observational or inadequately randomized trials. Therefore, previous studies have not presented sound evidence for judging the effect of improved water supply.

We thus undertook a community-based randomized intervention trial in a rural area of the Volta region in Ghana. This study aims to investigate the effect of source-based improved water supply on child diarrheal prevalence of children under five, employing a matched cluster randomized trial design to overcome the limitations previous studies have encountered. We also aim to produce critical information to provide a basis for a better approach to water quality interventions.

## 2. Methods

### 2.1. Study Setting

This is a cluster-randomized controlled trial on drilling or rehabilitating boreholes in the rural areas of Ghana. The Ghana Volta Region Water, Sanitation and Hygiene (WASH) project was carried out from March 2012 to December 2014, funded by the Korea International Cooperation Agency and implemented by World Vision Ghana.

The Krachi West and Krachi East districts, the target area, are situated on the northern shores of Lake Volta. The area is 400 km away from Accra to the Northeast and bounded by the Jasikan district to the southeast, Kadjebi district to the east, Nkwanta district to the northeast, East Gonja district to the north, and Sene district to the west. It covers an area of about 5658 km^2^, and according to the 2000 national census, its total population is 192,377. The inhabitants are mainly the Konkombas, Ewes, Akan, Nchumurus, and Krachi. Inadequate sources of water and the incidence of waterborne disease are major issues to be addressed in this area [29,30].

The intervention entailed drilling and rehabilitating boreholes, constructing latrines for schools and markets, and hygiene education and campaigns. A cluster design was chosen because the effect of boreholes was expected to be community-wide rather than at the individual level. The trial was carried out in rural communities without improved, clean water (with no boreholes or with broken boreholes) in two districts of the Volta Region in Ghana.

The District Assembly in each district provided a list of all communities in its district. Of the 557 communities located in the Krachi East and West districts, 165 target communities were selected for drilling or rehabilitating boreholes from this list. Communities whose participation in the study was considered infeasible were excluded (*i.e*., ones located in places too remote to reach, that were accessible only with difficulty, or that already had access to clean/safe water).

### 2.2. Implementation of Intervention

Of 106 target communities for drilling new boreholes, 78 (73.6%) boreholes were successfully drilled during the project period. In addition, 83 were rehabilitated in 59 communities. The intervention was successfully undertaken in all 10 intervention communities (100%).

Eighty-two communal latrines were constructed in markets or schools. A pumping test was conducted, and the boreholes were finally drilled when the test proved the capacity of producing 13.5 L/min. Water quality tests were conducted by the Ghana Standard Authority for assessing biochemical pathogens, strictly complying with WHO water quality standards. The hand-pump model of the borehole is the Afridev, which is a 100-mm diameter cylinder. In Krachi West, eight boreholes were tested and found to contain too much iron or manganese, and a borehole in one community was found to be contaminated with Arsenic; people were prohibited from using these boreholes. The project was composed of water, sanitation, and hygiene activities. Before the second round of the survey, the WASH committee members were trained in 68 communities in Krachi West and 44 communities in Krachi East for borehole management. A one-day community education program was conducted in 65 communities in Krachi West and 44 communities in Krachi East, once in each community for hand-washing practices, and for water and sanitation improvement. The education participants included 2245 male and 3085 female community members in Krachi West and 2345 males and 2458 females in Krachi East.

### 2.3. Study Design

Since the trial was based on a phased-in study design, boreholes were also drilled or rehabilitated in the other 10 communities under control arms. Borehole drilling or rehabilitation was completed in the control group immediately after the second round of the survey.

The second round of the survey started eight weeks after the intervention community had benefitted from the clean water supply by borehole drilling or rehabilitation. 

**Figure 1:**
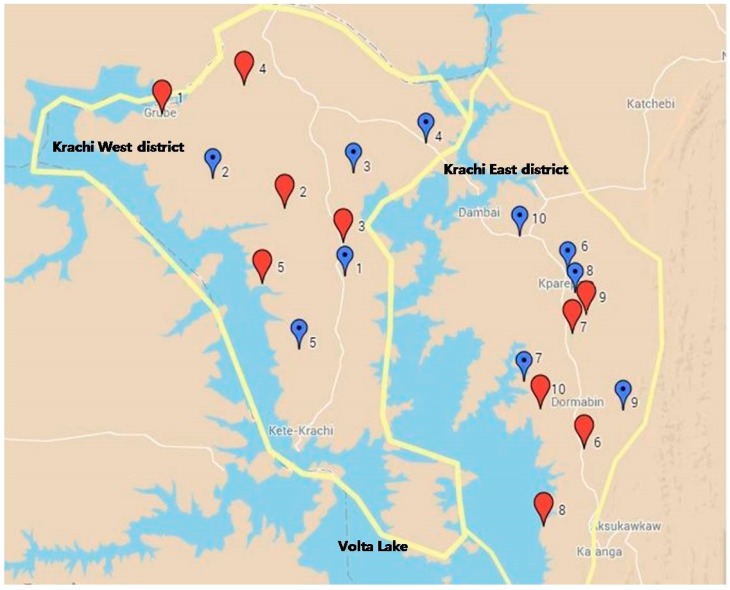
Geographical allocation of intervention and control communities. The yellow lines show the target districts. The numbered balloons represent the study communities of the matched pairs: blue for the intervention groups, red for controls. The white lines indicate the main roads in each district.

Figure 1 illustrates the geographical allocation of intervention and control communities in the Krachi West and Krachi East Districts of the Volta Region of Ghana. Among households with children under five, 607 were enrolled at the baseline survey.

Of the 165 target communities, 20 communities were selected by random sampling after stratifying by community population and geographical location. After selecting 20 communities, we matched similar communities, creating 10 community pairs. Pairing was conducted after the baseline survey, based on similar diarrhea prevalence among children under five, geographic location, population densities, and ethnic group distribution (Figure 1).

### 2.4. Randomization and Eligibility Criteria

**Figure 2:**
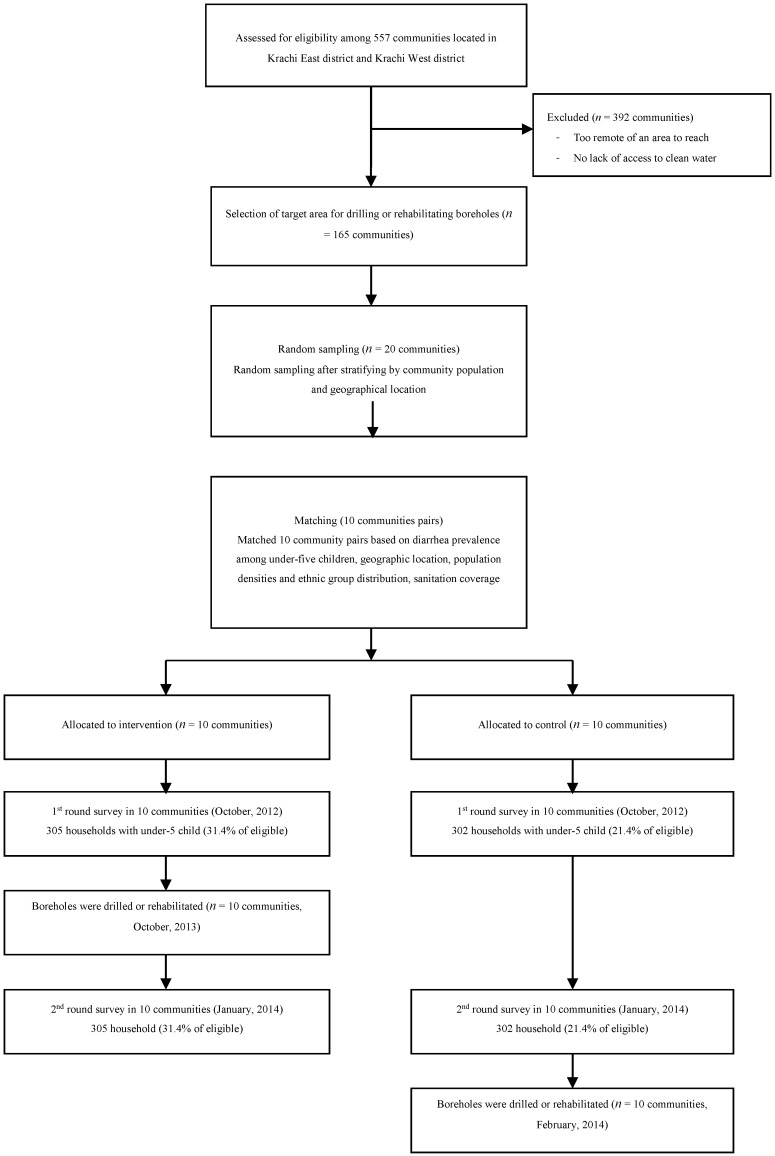
Study flow diagram.

We randomly allocated one cluster in each pair to either the intervention or control on the basis of a coin toss, which was conducted by community leaders during the community group meeting (Figure 2).

Mothers or caregivers having at least one child under five in her household were registered after agreeing to participate in the survey with written informed consent. The WASH project has been implemented for two years; but for exploring the effect of improved water access, the second round of the survey was undertaken two months after the completion of borehole drilling or rehabilitation in the intervention communities.

### 2.5. Primary End Point

The primary outcome measure is the 14-day prevalence of reported diarrhea in the household detected by parental report. 

### 2.6. Sample Size Calculation

The sample size for the primary endpoint, the period prevalence of diarrhea, was calculated using methods for cluster randomization trials [31]. Before conducting the baseline survey, existing data [29,30] from the study area was used to estimate the baseline prevalence of diarrhea (30%) as well as the within-community clustering (coefficient of variation, *k* = 0.3). In the 20 communities, 600 households were registered for the survey. The 20 communities achieved 85% power for a 15% reduction in prevalence and a 5%, two-sided significance level. The corresponding estimate of the design effect is 2.12. 

### 2.7. Data Collection

The 20 data collectors who made household visits comprised a teachers group, supervised by the project manager and independent supervisor. The survey was developed in English and translated into Ewe, the local language in the project area. Fluent speakers conducted the survey, which included demographics; educational level of household heads; household monthly income and expenditures; ownership of a household latrine; type of household water storage; days of water storage; total quantity of water used per day per person; water treatment practice; and hand washing practices. Data were recorded on the spot by tablet PCs for the household surveys. In addition to the administration of questionnaires, observations were concurrently conducted on hygiene practices and sanitation at the household and community level. For assessing compliance, we developed a questionnaire and asked whether a household was utilizing boreholes drilled or rehabilitated by the KOICA-World Vision project as their main water source.

The geographical location of each community was recorded by a handheld GPS for stratification purposes. In October 2012, a team of 20 field interviewers visited every potential member for the baseline survey, explained the study, asked for the mothers’ or caregivers’ consent, and completed an individual questionnaire and observation. Community members in the intervention group benefitted from the improved water supply of the project beginning in October 2013, while the control community benefitted beginning in February 2014. The second round of the survey was conducted in January 2014 to investigate the effect of source-based water intervention.

The interview questionnaire was developed by adapting the Demographic Health Survey and was piloted on the first day of the survey by the survey team. An independent observation followed after completing the interview at each household. The study was approved by the Ethics Committee of the Ghana Health Service (Approval Number: GHS-ERC 070114) and was conducted in close collaboration with the Service. A modified Poisson regression was used to estimate the prevalence ratio [32], which was the ratio of diarrhea prevalence in the intervention community that benefitted from borehole drillings or rehabilitation, relative to the control community. SAS 9.2 (SAS Institute Inc. Cary, North Carolina, CA, USA) was used for the analysis. All estimates are presented with 95% CIs. Intention-to-treat analysis was undertaken to assess the effects of water source improvement.

However, since some members of the community do not follow the protocol for their assigned treatment, the resultant treatment contamination can produce misleading findings, and thus “per protocol” and “as treated” analysis techniques were also used [33]. In per-protocol analysis, individuals are included in the analysis only if they followed the assigned protocol and are removed from the analysis entirely if they do not follow protocol. In as-treated analysis, all households are analyzed on the basis of the water supply ultimately received, regardless of the treatment to which they were randomly assigned [33]. This study is registered as an International Standard Randomized Controlled Trial (ISRCTN15191892).

## 3. Results

### 3.1. Baseline Characteristics

The basic characteristics of the intervention communities and controls are presented in Table 1. The randomization results indicate an appropriate balance between the arms by showing that the intervention and control groups are evenly distributed in terms of the key variables related to the main outcome. Except in sanitation coverage (*i.e.,* percentage of households with a latrine), there is no significant difference between the main indicator, diarrhea prevalence itself, or major risk factors, such as hand washing practices and types of water storage between the arms. 

### 3.2. Hand-Washing Practices after the Intervention

Table 2 clearly shows the balance in hand-washing behaviors between the intervention and control group was maintained after the project implementation except in “Hand-washing before feeding a child”. The percentage of caregivers practicing hand-washing before feeding a child became higher in the control group than in the intervention group, which might have led to underestimation of the prevalence ratio.

### 3.3. Diarrhea Prevalence in Intervention and Control Communities

**Figure 3:**
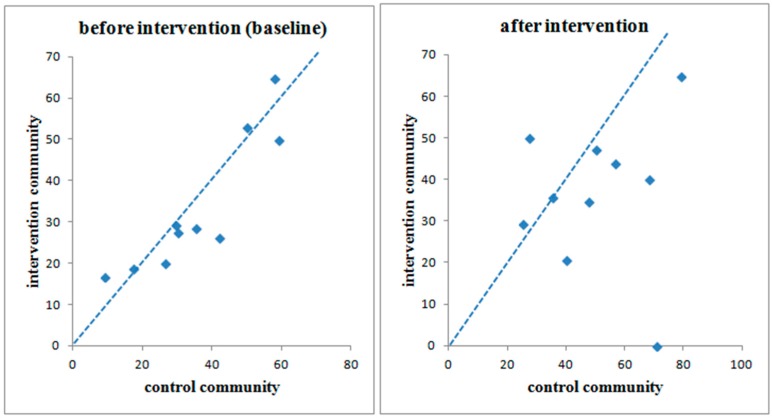
Diarrhea prevalence in intervention and control communities. If a dot is located below the line of equality, it means the diarrheal prevalence of the control community is higher than that of its counterpart in the pair. After the intervention, diarrheal prevalence fell much farther below the line of equality.

Figure 3 shows the within-cluster diarrhea prevalence for each of the 10 cluster pairs in the first and second round of the survey. The line of equality has been superimposed on this graph. In the majority of cluster pairs, diarrhea prevalence became much lower in the intervention communities after improved water was supplied. 

**Table 1:** Baseline Characteristics.

Variables	% Or Unit (Standard Deviation)	
	Control	Intervention
**Main Indicator**		
Prevalence of diarrhea	31.30%	27.90%
Age of head of household (years)	44.65 (14.97)	44.14 (14.61)
Level of Education		
Not educated or primary level not completed	60.10%	62.80%
Completed more than primary school	39.90%	37.20%
No. of children under five	1.64 (1.33)	1.48 (1.32)
Household expenditures per month (US $)	38.26 (36.82)	39.89 (43.95)
Household income per month (US $)	50.10 (48.49)	60.70 (94.49)
**Sanitation**		
Household toilet	16.90%	9.60%
Open defecation	56.70%	53.70%
Other (neighbor’s latrine, communal latrine)	26.4%	36.7%
**Water Storage**		
Storage tank	7.50%	4.70%
Barrel	67.80%	70.80%
Basin	7.50%	10.10%
Bucket	2.00%	1.00%
Gallon	8.20%	5.70%
**Container Type**		
Containers with lid, cover	68.20%	71.30%
Containers with tap	0.30%	0.70%
Narrow mouth (<10 cm), uncovered	4.80%	4.00%
Narrow mouth (>10 cm), uncovered	29.30%	30.60%
Average storage days	2.28 (2.10)	2.02 (1.46)
Total quantity of water per day per person (L)	22.61 (17.12)	19.52 (12.85)
Water treatment (no treatment)	88.00%	90.40%
**Hand Washing Practice**		
Before eating	94.80%	94.20%
After defecation	90.90%	87.80%
Before food preparation	34.80%	32.50%
After cleaning a child’s buttocks	7.00%	7.50%
Before feeding a child	2.80%	2.70%
After handling a sick person	3.10%	5.40%
After returning from a social gathering	8.70%	7.90%
Washing with soap	96.60%	97.00%
Knowledge of diarrhea	65.60%	61.00%

**Table 2:** Change in hand-washing practices.

Hand Washing Practices	Control Group		Intervention Group		*p* Value
	Baseline	After Intervention	Baseline	After Intervention	
Before eating	94.80%	74.40%	94.20%	74.50%	0.971
After defecation	90.90%	89.00%	87.80%	85.60%	0.225
Before food preparation	34.80%	40.90%	32.50%	44.10%	0.435
After cleaning a child’s buttocks	7.00%	23.10%	7.50%	21.90%	0.720
Before feeding a child	2.80%	19.90%	2.70%	13.70%	0.044
After handling a sick person	3.10%	4.00%	5.40%	4.70%	0.675
After returning from a social gathering	8.70%	8.90%	7.90%	8.50%	0.853
Washing with soap	96.60%	96.20%	97.00%	97.10%	0.530

### 3.4. Impact on Diarrhea Prevalence Following the Intention-to-Treat Analysis Method

Diarrhea prevalence at the baseline and second round of the survey are shown for each community in Table 3. At baseline, the diarrhea prevalence was 39.5% in the control group and 33.6% in the intervention group in the Krachi West district, and 31.1% and 27.9%, respectively, for each group in the Krachi East district. In both districts, the diarrhea prevalence increased up to 59.2% for the control and 44.7% for the intervention, and 34.7% and 27.5%, respectively, at the second round of the survey. Diarrhea prevalence varied between pairs both at the baseline and second round of the survey. The crude prevalence ratio for the diarrhea in the intervention communities compared with the controls was 0.85 (95% CI 0.74–0.97) for Krachi West, 0.96 (0.87–1.05) for Krachi East, and 0.91 (0.83–0.98) for both districts. Adjusting for sanitation coverage, which showed an imbalance between the two treatment arms at baseline, the prevalence ratio remained virtually unchanged (0.82, 0.71–0.96; 0.95, 0.86–1.04; 0.89, 0.82–0.97 in the same order).

**Table 3:** Effect of improved water supply (intention-to-treat analysis).

District	Paired Communities			Baseline		After Intervention		Result (Crude)		Result (Adjusted ^3^)	
	Pair	Control	Intervention	Control	Intervention	Control	Intervention	PR ^1^	CI ^2^	PR	CI
Krachi West	pair 1	Grubi	Ntewusae	20/76 (26.3%)	15/75 (20.0%)	43/76 (56.6%)	33/75 (44.0%)	0.88	0.76–1.03	0.81	0.68–0.96
	pair 2	Kpollo	Kaliako	11/19 (57.9%)	11/17 (64.7%)	15/19 (78.9%)	11/17 (64.7%)	0.92	0.48–1.76	0.90	0.44–1.84
	pair 3	Ankaase	Bakam	8/16 (50.0%)	9/17 (52.9%)	8/16 (50.0%)	8/17 (47.1%)	1.00	0.62–1.62	1.00	0.62–1.62
	pair 4	Majimaji	Papaye	13/22 (59.1%)	10/20 (50.0%)	15/22 (68.2%)	8/20 (40.0%)	0.66	0.41–1.07	0.64	0.40–1.04
	pair 5	Shitor Kope	Gyeasayor	8/19 (42.1%)	6/23 (26.1%)	9/19 (47.4%)	8/23 (34.8%)	0.79	0.56–1.12	0.70	0.45–1.09
	**Subtotal**			60/152 (39.5%)	51/152 (33.6%)	90/152 (59.2%)	68/152 (44.7%)	0.85	0.74–0.97	0.82	0.71–0.96
Krachi East	pair 6	Tokurano Attafie	Kparekpare	13/75 (17.3%)	14/75 (18.7%)	19/75 (25.3)	22/75 (29.3)	1.03	0.92–1.16	0.99	0.89–1.09
	pair 7	Nwane Akura	Tsikatakope	9/30 (30.0%)	8/29 (27.6%)	12/30 (40.0)	6/29 (20.7)	0.86	0.68–1.08	0.83	0.65–1.07
	pair 8	Adokwata Tornu	Okuma Akura	6/17 (35.3%)	4/14 (28.6%)	6/17 (35.3)	5/14 (35.7)	0.95	0.67–1.36	1.00	0.69–1.46
	pair 9	Abongo Akura	Katafua Junction	5/17 (29.4%)	5/17 (29.4%)	12/17 (70.6)	0/17 (0.00)	0.53	0.36–0.77	0.53	0.36–0.77
	pair 10	Atsigode Kope	Kwame Akura	1/11 (9.1%)	3/18 (16.7%)	3/11 (27.3)	9/18 (50.0)	1.15	0.89–1.48	1.15	0.89–1.48
	**Subtotal**			34/150 (22.7%)	34/153 (22.2%)	52/150 (34.7%)	42/153 (27.5%)	0.96	0.87–1.05	0.95	0.86–1.04
**Total**				94/302 (31.1%)	85/305 (27.9%)	142/302 (47.0%)	110/305 (36.1%)	0.91	0.83–0.98	0.89	0.82–0.97

^1 .^Prevalence ratio; ^2^ 95% confidence interval; ^3^ Sanitation was adjusted for in the model to remove the bias due to residual imbalance since it was not balanced even after randomization.

### 3.5. Compliance Rate

The second round of survey results shows that a considerable number of people did not comply with the study trial (Table 3). For instance, the compliance rate was only 57.9% in the Grubi community of the control arm, suggesting that 42.1% of people had benefitted from the clean water supply through the project, which could be possible since drilling or rehabilitating boreholes had been occurring in 145 additional communities apart from the target communities of this study. In addition, some of the community members in the intervention group were shown to have not benefitted from the intervention. The compliance rate was 65.1% for the control and 94.1% for the intervention communities in Krachi West, 65.3% and 74.5% in Krachi East, and 65.2% and 84.3% overall for each arm, respectively. On the basis of these results, we reanalyzed the impact after categorizing the respondents at the second round of the survey into the people who complied and did not comply under each intervention arm (people benefitting and not benefitting from the drilling or rehabilitating of boreholes under each intervention arm, both with and without combining across arms).

### 3.6. Per-Protocol and As-Treated Analyses

Restricting our investigation to the people who complied with the trial, the results also show the impact of providing a clean water supply by drilling or rehabilitating boreholes on the relative reduction in diarrheal prevalence of children under five (Table 4). Although the diarrheal prevalence increased in both the control and intervention communities, the results suggest that the increase was significantly different between the groups. The diarrheal prevalence among people in the control communities rose from 39.5% at baseline to 65.7% at the second round of the survey, while it increased from 33.6% to 44.8% in the intervention communities in the Krachi West district. Similarly, it rose from 22.7% to 40.0% for the control and from 22.2% to 26.3% in intervention communities in the Krachi East district. Overall diarrheal prevalence rose from 31.1% to 53.1% in the control and from 27.9% to 36.6% in the intervention group. According to the per-protocol analysis, the prevalence ratio for diarrhea in the intervention communities compared with the controls was 0.82 (95% CI 0.71–0.96) for Krachi West, 0.94 (0.85–1.04) for Krachi East, and 0.89 (0.81–0.97) for both districts. According to the as treated analysis, comparing the diarrheal prevalence between the people drinking clean water from boreholes and those not drinking regardless of their allocation to one arm or the other, the prevalence ratio for diarrhea of the beneficiaries compared with non-beneficiaries was 0.47 (95% CI 0.29–0.76) for Krachi West, 0.57 (0.35–0.94) for Krachi East, and 0.57 (0.41–0.80) for both districts together.

**Table 4:** Effect of water quality improvement (per-protocol and as-treated analyses).

District	Pair	Compliance				Diarrhea Prevalence				Per-Protocol		As Treated	
		Control		Intervention		Control		Intervention					
		Name	NO ^1^	Name	YES ^2^	Access	No	Access	No	PR ^3^	CI ^4^	PR	CI
Krachi West	pair 1	Grubi	44/76 (57.9%)	Ntewusae	70/75 (93.3%)	13/32 (40.6%)	30/44 (68.2%)	32/70 (45.7%)	1/5 (20.0%)	0.86	0.73–1.02	0.86	0.74–1.01
	pair 2	Kpollo	19/19 (100%)	Kaliako	13/17 (76.5%)	-	15/19 (78.9%)	8/13 (61.5%)	3/4 (75.0%)	0.97	0.49–1.93	0.90	0.46–1.74
	pair 3	Ankaase	1/16 (6.3%)	Bakam	17/17 (100%)	8/15 (53.3%)	0/1 (0.0%)	8/17 (47.1%)	-	1.44	0.80–2.60	1.21	0.65–2.27
	pair 4	Majimaji	16/22 (72.7%)	Papaye	20/20 (100%)	4/6 (66.7%)	11/16 (68.8%)	8/20 (40.0%)	-	0.67	0.41–1.11	0.70	0.43–1.16
	pair 5	Shitor Kope	19/19 (100%)	Gyeasayor	23/23 (100%)	-	9/19 (47.4%)	8/23 (34.8%)	-	0.79	0.56–1.12	0.79	0.56–1.12
	**Subtotal**	99/152 (65.1%)		143/152 (94.1%)		25/53 (47.17%)	65/99 (65.7%)	64/143 (44.8%)	4/9 (44.44%)	0.82	0.71–0.96	0.83	0.72–0.96
Krachi East	pair 6	Tokurano Attafie	56/75 (74.7%)	Kparekpare	61/75 (81.3%)	3/19 (15.8%)	16/56 (28.6%)	17/61 (27.9%)	5/14 (35.7%)	1.01	0.89–1.14	0.99	0.87–1.11
	pair 7	Nwane Akura	2/30 (6.7%)	Tsikatakope	23/29 (79.3%)	10/28 (35.7%)	2/2 (100%)	5/23 (21.7%)	1/6 (16.7%)	0.87	0.63–1.21	0.94	0.71–1.25
	pair 8	Adokwata Tornu	14/17 (82.4%)	Okuma Akura	7/14 (50.0%)	1/3 (33.3%)	5/14 (35.7%)	2/7 (28.6%)	3/7 (42.9%)	0.90	0.62–1.32	0.90	0.63–1.28
	pair 9	Abongo Akura	17/17 (100%)	Katafua Junction	11/17 (64.7%)	-	12/17 (70.6%)	0/11 (0.0%)	0/6 (0.0%)	0.53	0.36–0.77	0.60	0.45–0.81
	pair10	Atsigode Kope	9/11 (81.8%)	Kwame Akura	12/18 (66.7%)	0/5 (0.0%)	3/6 (50.0%)	6/12 (50.0%)	3/6 (50.0%)	1.08	0.83–1.42	1.03	0.80–1.33
	**Subtotal**	98/150 (65.3%)		114/153 (74.5%)		14/55 (25.45%)	38/95 (40.0%)	30/114 (26.3%)	12/39 (30.77%)	0.94	0.85–1.04	0.93	0.85–1.03
**Total**		197/302 (65.2%)		257/305 (84.3%)		39/108 (36.11%)	103/194 (53.1%)	94/257 (36.6%)	16/48 (33.33%)	0.89	0.81–0.97	0.90	0.83–0.98

^1^ Number of households not drinking from new or rehabilitated boreholes; ^2^ Number of households drinking new or rehabilitated boreholes; ^3^ Prevalence ratio; ^4^ Confidence interval; - means “not applicable because denominator is zero”.

## 4. Discussion

Supplying clean water by drilling or rehabilitating boreholes reduced diarrheal prevalence by 11% (CI 3%–18%) in our study. Our baseline data supports the comparability between the control and intervention groups; specifically, the baseline prevalence of diarrhea was very similar in each of the matched pairs. While an imbalance was found in sanitation coverage, it was adjusted for in the analysis, and the impact estimates remained virtually unchanged. Although there was a difference in the household income per month, the value of the household expenditures per month was very similar, and we thus concluded that income did not affect the diarrheal prevalence significantly in the context of remote rural Ghana.

Members of the communities in the control group were not informed that their communities were a comparison group, except for their community leaders who had participated in the selection procedure for the intervention arm by a coin toss. Borehole drilling or rehabilitation was undertaken concurrently in both intervention arms, but the final steps of completion were taken in the intervention group earlier than the control by eight weeks. Since the number of target beneficiary communities was 165, and completion dates varied across communities, it was possible for people to remain unaware of the arm in which they were included. Furthermore, the study hypothesis was not disclosed even though the general objectives were explained when obtaining informed consent. For this reason, we surmise that courtesy bias, or underreporting of diarrheal prevalence among the household members using the boreholes, was not severely induced in the study, even though we could not totally rule out the possibility. However, the study could not completely overcome the limitation of not blinding since people could easily tell whether their borehole was ‘operational’ at the time of the second round of the survey.

As described in the Results section, severe "contamination" was found to have taken place between the control and intervention arms, mainly resulting from the "spillover effect". Some people in control communities were thought to have travelled to neighboring communities, which were benefiting from borehole drilling or rehabilitation, to take advantage of the improved water supply. The study communities in our trial were carefully chosen with respect to their geographical location, allowing for large distances and long travelling periods between communities. However, borehole drilling or rehabilitation were also being undertaken in a number of communities not falling under any trial arm, perhaps motivating some of the control community members to travel to neighboring communities with functioning boreholes. We infer that the impact calculated according to the intention-to-treat model was considerably underestimated in this trial due to the spillover effect. 

Hygiene education and a campaign, as well as school and communal latrine construction, have also been undertaken during this project implementation, but these were not randomized. The results clearly demonstrate no significant difference between the two groups with regard to risk factors for diarrhea. Most of the indicators remained at a similar level in both groups, including sanitation coverage.

The one-day educational training was conducted once in each of the 20 communities of the study area. One latrine was constructed in Ntewusae (Intervention community); one in Kpollo (Control); one in Papaye (Intervention); three in Tokorano (Control); and three in Kparekpare (Intervention). In Tokorano and Kparekpare, the latrines were constructed after the study period. Even the number of latrines constructed was equal for the pair-matched communities. The communities where the latrines were constructed during the study period were Ntewusa, Kpollo, and Papaye. In Kpollo and Papaye, the latrines were constructed in schools. The diarrheal prevalence in the study was restricted to children under five years of age. We therefore suppose latrine construction in school might not have heavily impacted the prevalence in this group though we could not exclude the possibility of low transmission from their family members attending schools. In addition, it is not likely that the new two-seater latrine in Ntewusa Clinc in Ntewusa community notably influenced the diarrheal prevalence of under-five children in the community.

The most plausible explanation for the result is that 11% of the reduction in diarrhea prevalence is attributable to borehole drilling or rehabilitation. The most recent systematic review [19] reported an 11% reduction in the relative risk of diarrhea with an improved water supply. Our study shows consistent results with the systematic review. A reduction of some 17% in diarrhea risk was suggested to be associated with the use of an improved water supply for the LiST (Lives Saved Tool) program [18]. However, in reality, the effect of water quality improvement heavily depends on the pre-intervention water quality as well as the risk factors concerning diarrheal prevalence such as sanitation coverage and hygienic practices. This study adds to evidence that the effect of improved water highly varies depending on the pre-intervention context. 

A seven-day recall period is commonly used in diarrhea trials [34], and a shorter recall period has been recommended [35]. The limitation of this study is that we used a 14-day recall period, which might have increased the subjectivity of reporting. Another limitation of the study is one-time observation after the intervention, which hindered us from exploring the seasonal variation of the improved water effect, even though diarrheal prevalence highly varies between dry and rainy seasons [36]. Considering ethical issues, however, we had to reduce the delay time of providing the improved water supply to the control communities as short a period as possible. 

In the per-protocol analysis, individuals were included in the analysis only if they followed the assigned protocol and were removed from the analysis entirely if they did not follow the protocol. Per-protocol and as-treated analyses remove the benefit of randomization. Another limitation of per-protocol analysis is that we could not control the potential confounding factors such as differential characteristics of community members who had not complied with the protocol. Investigating the reasons some intervention community members did not use the boreholes and some control group members did access boreholes was outside the scope of our study. However, this would be a worthy object of investigation in a follow-up study.

We chose to record the period prevalence rather than the point prevalence because it can achieve a higher study power, although it could also bias the prevalence ratio towards no effect if the disease is common [37]. Diarrhea is the main killer in Ghana of both adults and children, but a number of people across this country still lack clean water. Across the world and in Ghana, integrated approaches, including hand washing and sanitation improvement, have been increasingly emphasized, but the importance of a clean water supply should not be underestimated. Expecting a substantial reduction in diarrhea from only behavioral change without the improvement of water sources cannot be viewed as a viable solution. This study emphasizes the significance of an improved water supply. 

## 5. Conclusions

Diarrhea is the main killer of children under-5 years, accounting for 9.2% of total child deaths [2] and the burden of diarrhea in children is disproportionately high in rural areas. We undertook a community-based randomized controlled trial to investigate the effect of source-based improved water supply on diarrheal prevalence of children under-5 in rural areas. Our results show that improved water supply could reduce diarrhea in under-5 children by 11%. The study presented sound evidence for judging the effect of improved water supply by applying rigorous methodology of a matched cluster randomized trial design. We hope that this study is used as a basis for comparison with the integrated WASH project in the future, which includes hand washing and/or sanitation improvement.
